# Capsinoids suppress fat accumulation via lipid metabolism

**DOI:** 10.3892/mmr.2014.2996

**Published:** 2014-11-24

**Authors:** QIN HONG, CHEN XIA, HU XIANGYING, YUAN QUAN

**Affiliations:** Department of Nutrition and Food Hygiene, School of Public Health, Central South University, Changsha, Hunan 410008, P.R. China

**Keywords:** capsinoids, adipocyte, lipid metabolism, fat accumulation, liver, adipose tissue

## Abstract

Capsaicin, found in red peppers, has been reported to have anti-obesity, anti-hypertension, anti-diabetes and anti-inflammatory functions. In the present study, we determined the effect of non-pungent capsinoids on the metabolism of adipocytes. We demonstrated that capsinoids suppressed fat accumulation *in vivo* and *in vitro* in mice. Liver, the main tissue of lipid metabolism, was treated by capsinoids, and HMG-CoA reductase, CPT-1, FAT/CD36 and GLUT4 were found to be increased significantly, which demonstrated promotion of the lipid metabolism in liver and adipose tissues. In addition, by adding capsinoids, the induced adipocytes also demonstrated significantly increased levels of HMG-CoA reductase, CPT-1, FAT/CD36 and GLUT4. Oil red O staining also demonstrated that capsinoids decreased fat accumulation in the adipocytes. In conclusion, these results indicate that capsinoids may be worth investigating as a potential cure for obesity.

## Introduction

It has been confirmed that red peppers demonstrate anti-obesity, anti-hypertension, anti-diabetes and anti-inflammatory functions (1). This activity infers that the peppers contain certain ingredients that play a crucial role in these processes. Following years of study, researchers have demonstrated that one of the most effective ingredients is capsaicin, which has a potential function in ameliorating insulin resistance (2). The receptor of capsaicin is transient receptor potential vanilloid subfamily member 1 (TRPV1) (3). By activating TRPV1, capsaicin participates in the mediation of noxious stress response (4,5). TRPV1 is a universal expression gene with abundant expression in adipocytes, β-cells, skeletal muscles and hepatocytes (6). Notably, capsaicin intake promotes energy consumption and fat metabolism, which has potential anti-obesity effects (7). This action may be a result of the effect of decreasing insulin resistance by capsaicin (8–10). In addition, capsaicin participates in type 2 diabetes. Several studies reveal that capsaicin has a modest effect in type 2 diabetes (11,12). Capsaicin has also demonstrated both beneficial and harmful effects on human health (4). In addition, capsaicin may act as a carcinogen and chemopreventive depending on different factors (13). Simultaneously, another component, non-pungent capsinoids, as an analog of capsaicin, may also activate TRPV1 (14). This molecule may exert some of the same effects as capsaicin, but without the harmful effects on human health. Thus, capsinoids demonstrate a potential medicinal value and are worthy of further study.

Adipocytokines secreted by fat tissue play an essential role in controlling the accumulation of fat in organisms, which is beneficial for health (15). Therefore, the upregulation of adipocytokine secretion to promote energy expenditure promises a potential way of preventing body fat accumulation (16). It has been reported that capsaicin stimulates energy expenditure via activation of adipocytokine secretion (2). In animals, capsaicin activates adipocytokine secretion, which induces catecholamine and uncoupling protein expression in brown adipose tissue (2). Thus, capsaicin induces energy expenditure by promoting oxygen consumption and core temperature. AMP-activated protein kinase (AMPK) is a key factor in this process, and is activated by lack of cellular energy. Once AMPK is activated, the glucose uptake and fatty acid oxidation in organisms are increased to produce more adenosine triphosphate (17). Although the capsaicin-inducing metabolism of adipocyte has been reported, the effects of non-pungent capsinoids on the metabolism of adipocytes have not been unveiled yet. This could provide potential anti-obesity, anti-hypertension and anti-diabetes activity and offer a new therapeutic strategy.

The aim of the present study was to elucidate the changes in cellular levels following capsinoid treatment in adiposity *in vivo* and *in vitro*, as well as the changes in HMG-CoA reductase, CPT-1, FAT/CD36 and GLUT4 expression that occur during the process.

## Materials and methods

### Mouse models of obesity

The high-fat diet (HFD) D12451 and low-fat diet (LFD) D12450 by Research Diets, Inc. (New Brunswick, NJ, USA) were formulated according to previous studies (18). Diets of HFD+5% capsinoids and LFD+5% capsinoids were also prepared. Five-week-old mice were fed with the diets for 12 weeks to induce obesity. Following this, the mice were weighed and the expression of HMG-CoA reductase, CPT-1, FAT/CD36 and GLUT4 was analyzed by semi-quantitative reverse transcription-polymerase chain reaction (RT-PCR) and western blotting. The study was approved by the ethics committee of Central South University, Changsha, China.

### Cell culture

The preadipocyte 3T3-L1 cells were first induced into adipose cells according to the previous study (19) and then divided into four groups: a non-treatment group, a low capsinoid treatment group (with 10^−10^ M capsinoids added), a medial capsinoid treatment group (with 10^−8^ M capsinoids added), and a high capsinoid treatment group (with 10^−6^ M capsinoids added). The addition of capsinoids was performed 24 h after the adipose cells had formed, as confirmed by microscopy. The cells were harvested 16 h after the addition of capsinoids. Following this, oil red O staining microscopic analysis was performed, and the expression of HMG-CoA reductase, CPT-1, FAT/CD36 and GLUT4 was analyzed by semi-quantitative RT-PCR and western blotting.

### Oil red O staining

The frozen tissue sections were prepared at a thickness of 8 mm. After fixing in formalin, the sections were washed in 60% isopropanol and then stained with freshly prepared oil red O working solution for 15 mins. Following rinsing with 60% isopropanol, the sections were analyzed under the microscope. Oil red O staining was also used to detect lipogenesis in cells.

### Semi-quantitative RT-PCR

The adipose tissues and cells were used to extract the total RNAs using TRIzol reagent (Invitrogen Life Technologies, Carlsbad, CA, USA). Next, the RNAs were reverse transcribed into first-strand cDNA by SuperScript II reverse transcriptase (Life Technologies, Gaithersburg, MD, USA) from 1 μg RNA. The primers were designed according to the sequences in GenBank (Table I). Semi-quantitative RT-PCR was performed on an Applied Biosystems 7500 real-time PCR system (Life Technologies). The reaction conditions were 1 cycle at 94°C for 4 min; 40 cycles at 94°C for 30 sec, 60°C for 30 sec and 72°C for 30 sec. The specifics of the primers were determined by dissociation curve. Following the reaction, the data were analyzed using SDS 1.3 software on the Applied Biosystems 7500 real-time PCR system.

### Western blotting

The tissues and cells were lysed in RIPA buffer and then centrifuged at 15,000 rpm for 15 min to obtain the supernatant. Subsequently, the extracted protein samples were resolved by 12% SDS-PAGE and transferred onto PVDF membranes (Amersham Biosciences, Piscataway, NJ, USA). After blocking in 4% skimmed milk for 10 min, the protein samples were probed by primary antibodies.

The primary antibodies of HMG-CoA reductase (ab98018), CPT-1 (ab128568), FAT/CD36 (ab78054) and GLUT4 (ab654) were purchased from Abcam (Cambridge, MA, USA. Then, secondary antibodies conjugated with horseradish peroxidase were incubated with the membranes. The signals were detected using the chemiluminescence system SuperSignal West Pico Chemiluminescent Substrate (Pierce, Rockford, IL, USA).

### Statistical analysis

All the data are shown as the means ± SEM of independent experiments. One-way analysis of variance was used to determine differences among groups. P≤0.05 was considered to indicate a statistically significantly difference.

## Results

### Capsinoids decrease fat growth

**Figure 1:**
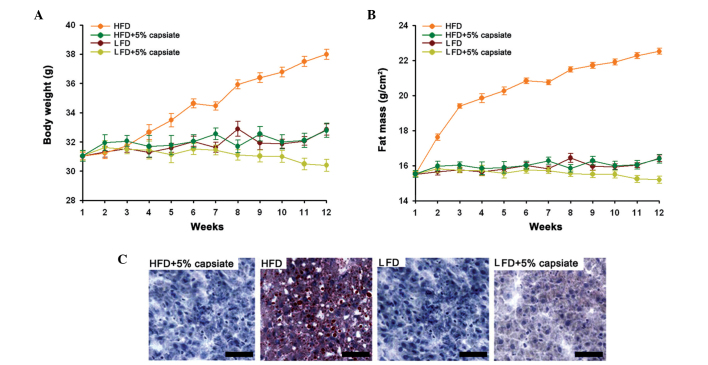
Capsiate decreases fat growth in mouse models of obesity. (A) Body weight changes in mice fed with a high-fat diet (HFD), HFD+5% capsinoids, low-fat diet (LFD) and LFD+5% capsinoids. (B) Fat mass changes in mice fed with HFD, HFD+5% capsinoids, LFD and LFD+5% capsinoids. (C) Oil red O staining of adipose tissue demonstrates the fat accumulation changes after feeding with HFD, HFD+5% capsinoids, LFD and LFD+5% capsinoids.

We first induced mouse models of obesity and assayed the body growth and fat growth. The results demonstrated that after feeding with the HFD, the body weight increased significantly compared with the other groups (Fig. 1A). Mice in the LFD+5% capsinoids group had the lowest body weight among all the tested groups. Similarly, the fat mass was the higher in the HFD group than in the other tested groups (Fig. 1B). In addition, the tissue sections also confirmed that the capsinoids decreased fat growth in liver tissue as they demonstrated significant oil red O signals while other groups had almost no fat accumulation in the tissues (Fig. 1C).

### Capsinoids induce lipid metabolism in liver

**Figure 2:**
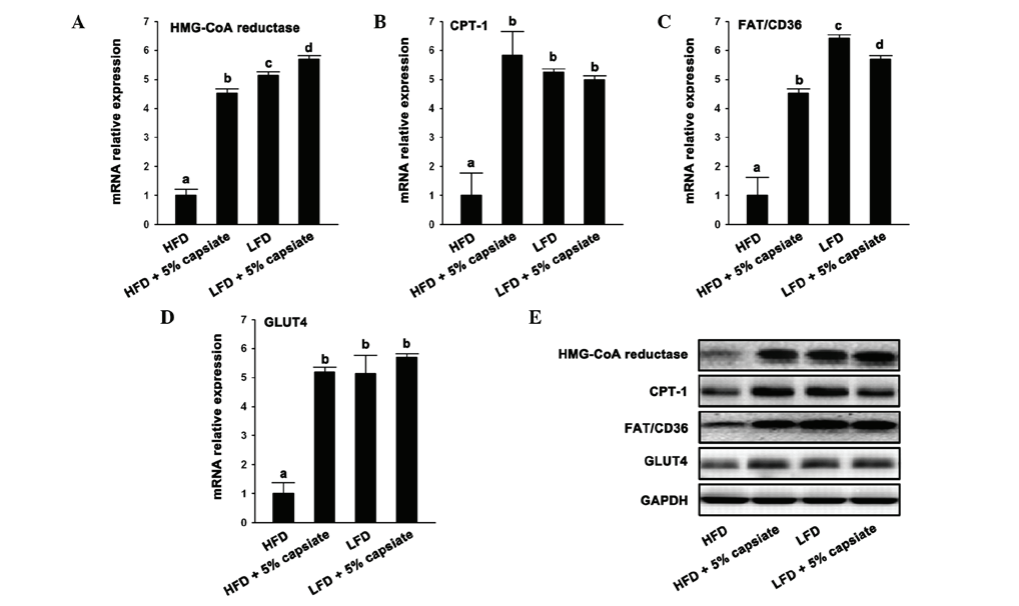
Capsinoids induce expression of lipid metabolism genes in liver, including HMG-CoA reductase, CPT-1, FAT/CD36 and GLUT4. (A–D) mRNA expression by semi-quantitative reverse transcription-polymerase chain reaction after feeding with a high-fat diet (HFD), HFD+5% capsinoids, low-fat diet (LFD) and LFD+5% capsinoids. The different characters indicate a significant difference among the groups (P<0.05). (E) Protein levels by western blotting after feeding with HFD, HFD+5% capsinoids, LFD and LFD+5% capsinoids.

Subsequently, the changes in the key genes which participate in lipid metabolism, including HMG-CoA reductase, CPT-1, FAT/CD36 and GLUT4, were assayed in the liver of obese mouse models. The results revealed that, for all the studied genes, the HFD group had the lowest mRNA expression (Fig. 2A–D) as well as protein expression (Fig. 2E). The HFD+5% capsinoids group demonstrated an increase in HMG-CoA reductase, CPT-1, FAT/CD36 and GLUT4 expression levels, which suggested that 5% capsinoid diets may promote lipid metabolism in liver.

### Capsinoids induce lipid metabolism in adipose tissue

**Figure 3:**
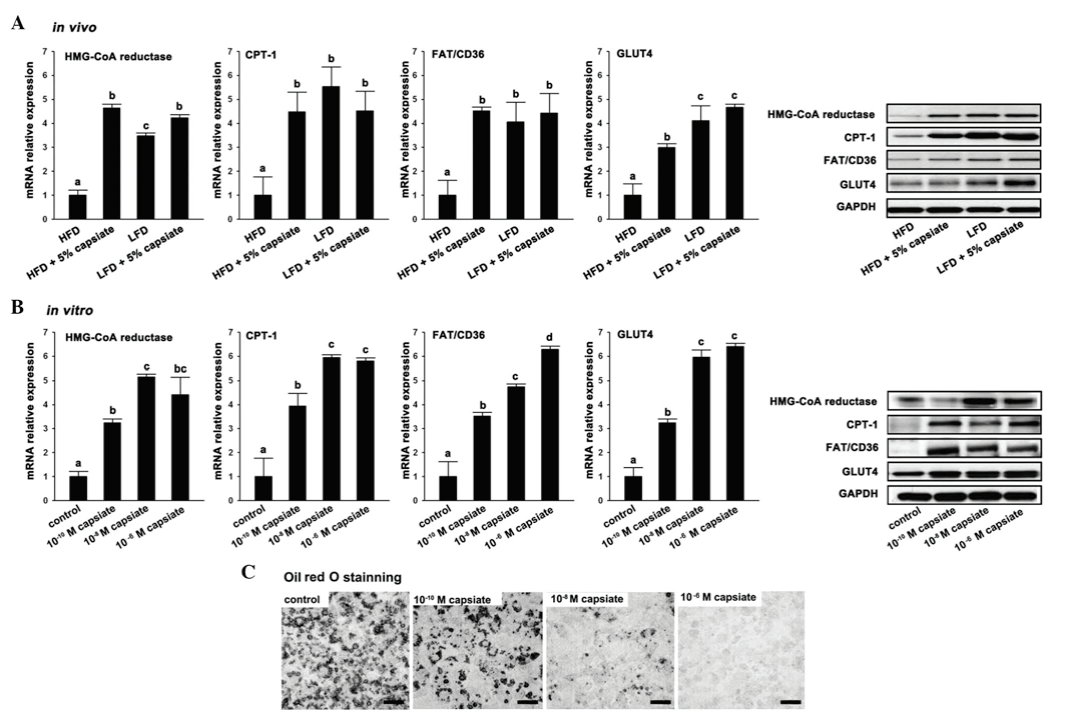
Capsinoids induce expression of lipid metabolism genes in adipose tissue (A) and induced adipose cells (B), including HMG-CoA reductase, CPT-1, FAT/CD36 and GLUT4. (C) Oil red O staining of adipose tissue indicates the fat accumulation changes following treatment with various concentrations of capsinoids.

We also analyzed the lipid metabolism in adipose tissue. The results revealed that all the tested genes had the lowest mRNA expression as well as protein expression in the HFD group (Fig. 3A), which indicated that the 5% capsinoid diets increased lipid metabolism. The *in vitro* study also supported this finding. In induced adipocytes, by adding capsinoids, the expression of HMG-CoA reductase, CPT-1, FAT/CD36 and GLUT4 increased significantly compared with the control group (Fig. 3B). In addition, oil red O staining also indicated that 10^−10^ M capsinoids decreased fat accumulation significantly in the adipocytes (Fig. 3C).

## Discussion

In the present study, we demonstrated that capsinoids have an inhibitory effect on fat accumulation which is due to the increasing effect of capsinoids on the lipid metabolism in liver and adipose tissues. Capsinoids, as an analog of capsaicin, also exhibited similar effects to capsaicin (20). However, capsinoids have a great advantage over capsaicin as they are non-pungent (21). It has been reported that capsinoids induce immune responses, having anti-inflammatory and antiproliferative effects on T cells (22). In addition, capsinoids are able to stimulate an antioxidant effect. It has also been reported by Haramizu *et al* (23) that after 2 weeks of treatment with capsinoids, human body fat accumulation was suppressed. In the present study, we also indicated that following intake of capsinoids, the body weight and fat mass index were suppressed even when feeding with a HFD. Thus, the mechanism of the suppression effect was also illustrated in the present study.

We then elucidated the expression changes of the lipid metabolism in liver and adipose tissues following treatment with capsinoids for various genes including HMG-CoA reductase, CPT-1, FAT/CD36 and GLUT4. All of these genes were significantly increased in liver and adipose tissues. These results suggested that the lipid metabolism was also upregulated by capsinoid treatment. HMG-CoA reductase is a rate-controlling enzyme which participates in the mevalonate pathway and is responsible for producing cholesterol and other isoprenoids. In normal cells of animals, HMG-CoA reductase is suppressed and degraded by low-density lipoprotein (24). In addition, HMG-CoA reductase has been considered to play a role in cholesterol synthesis and demonstrated activity in lipid metabolism (25). CPT1 is an essential enzyme in the beta-oxidation of long-chain fatty acids which participates in fatty acid activation and oxidization within the mitochondrial matrix (26. FAT/CD36 has functions in long-chain fatty acid uptake and could be irreversibly inhibited by sulfo-N-succinimidyl oleate, which is associated with myocardial fatty acid uptake (27). GLUT4 is a insulin-regulated glucose transporter which was identified in adipose tissues and striated muscle and has demonstrated a facilitated diffusion of circulating glucose in fat cells (28). Thus, these genes are significant components in lipid metabolism. In liver, the upregulated genes provide an indication that, following intake of capsinoids, the lipid metabolism pathway is significantly stimulated. The same effect was also observed in adipose tissues. In mice, it has been reported that capsinoids upregulate uncoupling protein in skeletal muscle and brown adipose tissue, which indicates that capsinoids play a notable role in energy expenditure, body weight and thermoregulation (29). A previous study has revealed that capsinoids suppressed body fat accumulation and raised oxygen consumption in the same way as exercise (29). Our findings correspond with these studies. However, the present study provides new evidence of the role of capsinoids in lipid metabolism.

It has been reported that capsaicin stimulated UCP1 expression in brown adipose tissue as well as in the concentration of serum adrenaline, which induced a depression of perirenal adipose tissue (30). Capsinoids are non-pungent, but have a similar function to capsaicin in stimulating UCP1 expression, resulting in changes in energy metabolism, adrenaline release and body fat accumulation. Thus, studies of capsinoids may provide information to support their potential application in decreasing fat. Capsaicin also demonstrated physiological effects on adrenaline release and increase in body temperature. These results also confirm that the metabolic effects of capsinoids are the same as those of capsaicin. Obesity occurs due to the imbalance between energy intake and consumption, which leads to weight gain and abdominal adipose tissue accumulation. Faraut *et al* demonstrated that capsinoid-induced UCP3 and UCP3 expression is a causative factor of weight loss (31). We have demonstrated in the present study that capsinoid intake reduced abdominal fat mass. Thus, it was also confirmed that capsinoids stimulate the lipid metabolism.

In conclusion, the results of the present study indicated that capsinoid intake stimulates fat metabolism, which may lead to a reduction in fat accumulation. The present findings suggest that compound capsinoids may be worth investigating as a cure for obesity.

## Figures and Tables

**Table I:** Primers used in this study.

Primer name	Sequences (5′ to 3′)
HMG-CoA reductase-forward	5′-TTCTGGCAGTCAGTGGGAACT-3′
HMG-CoA reductase-reverse	5′-TCCTCGTCCTTCGATCCAA-3′
CPT-1-forward	5′-GGACAGAGACTGTGCGTTCCT-3′
CPT-1-reverse	5′-GCGATATCCAACAGTGCTTGA3′
FAT/CD36-forward	5′-GATGACGTGGCAAAGAACAG-3′
FAT/CD36-reverse	5′-TCCTCGGGGTCCTGAGTTAT-3′
GLUT4-forward	5′-CTTCATCATTGGCATGGGTTT-3′
GLUT4-reverse	5′-AGGACCGCAAATAGAAGGAAGA-3′
